# Precision health diagnostic and surveillance network uses *S* gene target failure (SGTF) combined with sequencing technologies to track emerging SARS‐CoV‐2 variants

**DOI:** 10.1002/iid3.634

**Published:** 2022-05-11

**Authors:** Rafael Guerrero‐Preston, Vanessa Rivera‐Amill, Karem Caraballo, Sebastian Rodríguez‐Torres, Ana Purcell‐Wiltz, Andrea A. García, Raphael S. Torres, Fernando T. Zamuner, Claudio Zanettini, Matthew J. MacKay, Rachel Baits, Daisy Salgado, Gaurav Khullar, Jessica Metti, Timothy Baker, Joel Dudley, Keilyn Vale, Gabriela Pérez, Lorena De Jesús, Yaima Miranda, Denise Ortiz, Amanda García‐Negrón, Liliana Viera, Alberto Ortiz, Jorge A. Canabal, Josefina Romaguera, Ivonne Jiménez‐Velázquez, Luigi Marchionni, José F. Rodríguez‐Orengo, Adriana Baez, Christopher E. Mason, David Sidransky

**Affiliations:** ^1^ LifeGene‐Biomarks, Inc San Juan Puerto Rico; ^2^ Center for Research Resources Ponce Health Sciences University‐Ponce Research Institute Ponce Puerto Rico; ^3^ University of Pittsburgh School of Medicine Pittsburgh Pennsylvania USA; ^4^ Biology Department University of Puerto Rico Río Piedras Puerto Rico; ^5^ Department of Otolaryngology‐Head and Neck Surgery Johns Hopkins University School of Medicine Baltimore Maryland USA; ^6^ Department of Pathology and Laboratory Medicine, Weill Cornell Medicine Cornell University New York New York USA; ^7^ Tempus Labs, Inc Chicago Illinois USA; ^8^ Neurology Medicine Department Palmetto General Hospital Miami Florida USA; ^9^ Department of Surgery University of Puerto Rico School of Medicine San Juan Puerto Rico; ^10^ Internal Medicine Department University of Puerto Rico School of Medicine San Juan Puerto Rico; ^11^ Obstetrics and Gynecology Department University of Puerto Rico School of Medicine San Juan Puerto Rico; ^12^ Biochemistry Department University of Puerto Rico School of Medicine San Juan Puerto Rico; ^13^ Otolaryngology Department University of Puerto Rico School of Medicine San Juan Puerto Rico

**Keywords:** algorithms, genomics, population surveillance, precision medicine, public health, SARS‐CoV‐2

## Abstract

**Introduction:**

The severe acute respiratory syndrome coronavirus 2 (SARS‐CoV‐2) pandemic revealed a worldwide lack of effective molecular surveillance networks at local, state, and national levels, which are essential to identify, monitor, and limit viral community spread. SARS‐CoV‐2 variants of concern (VOCs) such as Alpha and Omicron, which show increased transmissibility and immune evasion, rapidly became dominant VOCs worldwide. Our objective was to develop an evidenced‐based genomic surveillance algorithm, combining reverse transcription polymerase chain reaction (RT‐PCR) and sequencing technologies to quickly identify highly contagious VOCs, before cases accumulate exponentially.

**Methods:**

Deidentified data were obtained from 508,969 patients tested for coronavirus disease 2019 (COVID‐19) with the TaqPath COVID‐19 RT‐PCR Combo Kit (ThermoFisher) in four CLIA‐certified clinical laboratories in Puerto Rico (*n* = 86,639) and in three CLIA‐certified clinical laboratories in the United States (*n* = 422,330).

**Results:**

TaqPath data revealed a frequency of *S* Gene Target Failure (SGTF) > 47% for the last week of March 2021 in both, Puerto Rico and US laboratories. The monthly frequency of SGTF in Puerto Rico steadily increased exponentially from 4% in November 2020 to 47% in March 2021. The weekly SGTF rate in US samples was high (>8%) from late December to early January and then also increased exponentially through April (48%). The exponential increase in SGFT prevalence in Puerto Rico was concurrent with a sharp increase in VOCs among all SARS‐CoV‐2 sequences from Puerto Rico uploaded to Global Influenza Surveillance and Response System (GISAID) (*n* = 461). Alpha variant frequency increased from <1% in the last week of January 2021 to 51.5% of viral sequences from Puerto Rico collected in the last week of March 2021.

**Conclusions:**

According to the proposed evidence‐based algorithm, approximately 50% of all SGTF patients should be managed with VOCs self‐quarantine and contact tracing protocols, while WGS confirms their lineage in genomic surveillance laboratories. Our results suggest this workflow is useful for tracking VOCs with SGTF.

## Graphical abstract

The evidence‐based Molecular Epidemiology and Genomic Surveillance algorithm, developed in this study to quickly identify emerging Variants of Concern (VOCs), is a valuable tool for identifying individual carriers of highly infectious variants with the *S* Gene Target Failure (SGTF) feature, such as Alpha and Omicron, who can then be effectively triaged for isolation, contact tracing, and treatment purposes.

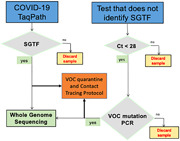

## 1 INTRODUCTION

The 2002 outbreak of severe acute respiratory syndrome coronavirus (SARS‐CoV), the first global pandemic of the 21st century, quickly spread from continent to continent.^1^ More than 8000 infections were initially reported with a 10% mortality rate. The SARS‐CoV pandemic demonstrated the importance of real‐time information in a rapidly progressing epidemic with a large number of cases and the need for frequent patient updates.^2^ The coronavirus disease 2019 (COVID‐19), caused by severe acute respiratory syndrome coronavirus 2 (SARS‐CoV‐2) was more infectious than SARS‐CoV and quick overwhelmed private and public health‐care systems worldwide.^3^ SARS‐CoV‐2 revealed the need to develop molecular and genomic epidemiology tools to track the public and population health impact of SARS‐CoV‐2 community spread.^4^

The lack of accurate real‐time SARS‐CoV‐2 data created massive challenges to local and national viral infection containment efforts. A variety of constantly changing pandemic directives to limit viral spread were put in place worldwide, without the benefit of real‐time public health surveillance information. Most countries opted for entry and exit health screening among international travelers and implementation of home quarantine, as tools to interrupt SARS transmission. The majority of initial COVID‐19 directives were not evidenced‐based. Confusing and sometimes contradictory guidelines eventually led to strong resistance from the business community and fueled the anger of citizens who felt their individual rights were being trampled on. Practical and legal challenges were encountered as pandemic directives and quarantine measures were put in place.

Local, State, and National Health Departments staff were overwhelmed by the daunting number of tasks required of them: SARS surveillance and case reporting; accurate, timely information and guidance; investigation and management of possible cases; tracking and quarantine of contacts; health risk assessment and communication; and infection control coordination with health‐care facilities. The need of a well‐coordinated genomic surveillance network to track viral spread and evolution, became apparent during the initial months of the pandemic.

As of April 22, 2022, more than 500 million cases and over 6 million deaths have been reported since the beginning of the SARS‐CoV‐2 pandemic in January 2020, and more than 11 billion vaccine doses have been administered to combat this viral infection (https://coronavirus.jhu.edu). The SARS‐CoV‐2 variant of concern (VOC), known as Alpha variant or B.1.1.7 (20I/501Y.V1, VOC 202012/01) detected in the United Kingdom in November 2020, was the first VOC of concern to quickly spread worldwide.^5^, ^6^, ^7^ The Alpha variant possesses many nonsynonymous substitutions of biological/immunological significance, in particular Spike mutations HVΔ69‐70, N501Y, and P681H, as well as ORF8 Q27stop and ORF7a.^6^, ^8^, ^9^ Alpha showed increased transmissibility and rapidly became the first dominant VOC in the United States during the second week of March 2021 (https://covid.cdc.gov).^10^, ^11^, ^12^, ^13^ Since then, the Delta (B.1.617.2) and Omicron (B.1.1.529) variants have replaced the Alpha variant as the dominant VOC in the United States and worldwide.^14^ The HVΔ69‐70 mutation initially found in the Alpha variant is a permissive mutation in the SARS‐CoV‐2 21765‐21770 genome region that removes Spike amino acids 69 and 70, which increases infectivity by allowing the acquisition of otherwise deleterious immune escape mutations.^15^ SARS‐CoV‐2 variants carrying the ∆H69/∆V70 deletion in the amino‐terminal domain of the Spike protein have emerged independently in at least six additional lineages of the virus: B.1.1.298, B.1.160, B.1.177, B.1.258, B.1.375, B.1.1.529.^16^

Detection of SARS‐CoV‐2 infection using a PCR‐based method is the gold standard for molecular diagnosis. The HVΔ69‐70 causes target failure in the TaqPath COVID‐19 reverse transcription polymerase chain reaction (RT‐PCR) Combo Kit (ThermoFisher) assay, catalog number A47814 (TaqPath).^17^ TaqPath is designed to coamplify sections of three SARS‐CoV‐2 viral genes: Nucleocapsid (*N*), Open Reading Frame 1ab (*ORF1ab*), and Spike (*S*).^18^ The Spike HV∆69/70 deletion prevents the oligonucleotide probe from binding its target sequence, leading to what has been termed *S* gene dropout or *S* gene target failure (SGTF).^5^ SGTF is associated with significantly higher viral loads in samples tested by TaqPath.^18^ *S* gene target late amplification (SGTL) has also been observed in a subset of samples having Cycle threshold^19^ values for *S* gene >5 units higher than the maximum *Ct* value obtained for the other two assay targets: *N* and *ORF1ab*.

The United States and countries where Alpha rapidly became the dominant SARS‐CoV‐2 variant required immediate and decisive action to minimize COVID‐19 morbidity and mortality.^12^, ^13^ However, at the time of the spread of the Alpha variant, the United States did not have a national genomic surveillance program with whole‐genome sequencing (WGS) capability in place. Therefore, only a small fraction of all new SARS‐CoV‐2 cases were sequenced ad‐hoc. SGTF has been shown to correlate with the Δ69‐70 mutation highly. Consequently, SGTF was used as a proxy to monitor SARS‐CoV‐2 lineage prevalence and geo‐temporal distribution, as well a near‐direct measure of Alpha and other SGTF‐positive variants.^17^, ^20^ Although some VOCs do not have this feature, the rapidly spreading Omicron VOC has also been shown to be strongly associated with SGTF.^21^, ^22^ Lessons learned from previously SGTF‐positive variant Alpha for which we were able to follow its epidemiologic course, are now informing current strategies for highly infectious VOCs such as Omicron.

In an urgent response to the SARS‐CoV‐2 global pandemic, a consortium of researchers and scientists working in academia, industry, and clinical laboratories implemented a Precision Health Diagnostic and Surveillance Network (PHx) in March 2020. PHx's original objective was to augment SARS‐CoV‐2 molecular testing capacity and implement a genomic surveillance network in Baltimore, New York, and Puerto Rico.^23^ The present work describes the development of an evidenced‐based genomic surveillance algorithm that combines RT‐PCR and sequencing technologies to identify highly infectious VOCs. This algorithm is a powerful tool to curb the spread of SARS‐CoV‐2 VOCs, enabling a reduction in illness, hospitalization, death, and postacute sequelae of COVID‐19 infection.^24^, ^25^, ^26^

## 2 MATERIALS AND METHODS

### 2.1 Precision health diagnostic and surveillance network

The PRECEDE/PROCEED Model (PPM)^27^ was selected to provide the evaluation framework for PHx conceptualization and implementation (Supporting Information: Figure 1). Weekly remote meetings began in March 2020 to perform Social, Epidemiological, Educational, Behavioral, and Environmental assessments in New York, Puerto Rico, and Baltimore, using the NIH I‐Corps Program framework.^28^ School of Medicine faculty from the University of Puerto Rico in San Juan, Johns Hopkins University in Baltimore and Weill Cornell in New York City were involved in the conceptualization and implementation of PHx. LifeGene‐Biomarks coordinated the PHx consortium and led the Baltimore initiative, while the Center for Puerto Rican Studies of Hunter College led the New York group, and the Puerto Rico Public Health Trust (PRPHT) led the Puerto Rico initiative.

## 3 RESEARCH SAMPLES COLLECTION

A team of investigators from the Medical Sciences Campus of the University of Puerto Rico (MSC), Johns Hopkins University School of Medicine, and LifeGene‐Biomarks obtained Institutional Review Board (IRB) approval from the University of Puerto Rico Medical School Institutional Review Board (IRB2770120) for a COVID‐19 biomarker development study, designed to validate the TaqPath COVID‐19 assay in paired, self‐collected nasal swab, saliva, and urine samples. Written informed consent was provided by the participants of the study. Paired samples from 280 participants were obtained from June to August 2020. Research samples were used to validate RT‐PCR and isothermal (LAMP) COVID‐19 clinical Laboratory Developed Tests in LifeGene‐Biomarks' laborator in Puerto Rico, following COVID‐19 Emergency Use Authorization (EUA) protocols submitted to the US Food and Drug Administration (FDA) for TaqPath. Deidentified data were used for this analysis.

## 4 CLINICAL SAMPLES COLLECTION

Deidentified data were obtained from 508,969 patients tested for COVID‐19 in clinical laboratories: 86,639 test results were from Puerto Rico and 422,330 test results were from samples collected in Connecticut, Illinois, New Jersey, and New York.

## 5 TAQPATH RT‐PCR ASSAY FOR DETECTION OF SARS‐COV‐2

Routine clinical COVID‐19 diagnostic testing was performed in Clinical Laboratory Improvement Amendments of 1988 (CLIA) regulated laboratories at University of Puerto Rico Medical Sciences Campus, Laboratorio Villa Ana, Inno Diagnostics Reference Laboratory, LifeGene‐Biomarks Laboratory, Yale University, Yale New Haven Hospital, and Tempus Labs, all following the EUA protocol for TaqPath COVID‐19. SGTF results were defined as any SARS‐CoV‐2‐positive sample with *N* or *ORF1ab C*_t_ < 30 and *S* gene undetermined. The data were aggregated on weekly and monthly totals for surveillance purposes.

## 6 SARS‐COV‐2 WGS

SARS‐CoV‐2 WGS was performed in a subset of samples from Puerto Rico in Ponce Research Institute. Briefly, SARS‐CoV‐2 WGS was completed using the Trio RNA Seq kit (Nugen Technologies) without using any human RNA depletion protocol. SARS_CoV‐2 RNA purification from nasopharyngeal specimens was done using the MagMAX™ Viral RNA Isolation Kit (Thermo Fisher Scientific) following the 200 μl purification protocol suggested for processing clinical specimens in the TaqPath™ COVID‐19 Combo Kit Emergency Use Authorization. The only modification made to the protocol was the omission of the bacteriophage MS2 addition to the samples since this is a shotgun approach and competing RNA can hamper such samples' sequencing. Following RNA extraction, the samples were quantified using a Qubit 2.0 with the Qubit™ RNA HS Assay Kit (Thermo Fisher Scientific). Since the Trio RNA Seq kit can process samples in the range of 500 pg and 50 ng and none of the selected samples exceeded 2.5 ng/µl no dilution was performed. Ten microliters from the RNA extraction were used to prepare the libraries. All steps for library construction were performed according to the manufacturer's protocol. The resulting libraries were quantified using the Qubit 2.0 and the Qubit™ dsDNA HS Assay Kit and diluted to a concentration of 4 nM with Tris buffer. Five microliters of the diluted libraries were pooled. A final dilution to 6 pM of the libraries with a 12% PhiX spike was heat‐denatured and loaded to a MiSeq instrument (Illumina). The MiSeq run was preformed using a six hundred cycles V3 kit at 2 × 251 cycles. After the run, MiSeq data were analyzed using Fastqc. Fastq files were trimmed off illumina adaptor sequences and quality filtered using Trim_Galore! (https://www.bioinformatics.babraham.ac.uk/projects/trim_galore/) with the following options: paired, q 30, illumina. After the quality trimming steps, each specimen fastq file was aligned to the NC_045512.2 SARS_CoV‐2 reference genome using Bowtie2^29^ very‐sensitive mode. The resulting SAM file was converted to a BAM file, sorted, and indexed using the samtools package.^19^ The resulting alignments were inspected using Tablet^30^ for Indel verification purposes. Then, a consensus sequence was generated from the BAM file using samtools mpileup, bcftools,^19^ and ivar^31^ packages. The resulting consensus was analyzed using the GISAID CoVsurver: Mutation Analysis of hCoV‐19 application for mutation screening and clade classification.

WGS data from 43,202 viral samples from Connecticut (*n* = 3,492), Illinois (*n* = 7,177), New Jersey (*n* = 5,058), New York (*n* = 27,020), and Puerto Rico (*n* = 461), analyzed using the CoVsurver: Mutation Analysis of hCoV‐19 application for mutation screening and clade classification, was downloaded on April 18, 2021, from the Global Influenza Surveillance and Response System (GISAID) site Most of the 43,202 samples reported in GISAID were sent for processing at Center for Disease Control (CDC) regional laboratories, using Illumina or nanopore sequencers. Sample‐specific WGS information can be obtained in https://www.gisaid.org.

## 7 SANGER SEQUENCING

A subset of SARS‐CoV‐2‐positive clinical samples with an SGTF were selected for Sanger Sequencing in Inno Diagnostics Laboratory. Sanger sequencing primers were designed to amplify and sequence two regions of interest from the SARS‐CoV‐2 genome between nucleotides 21600 and 23200 of the Spike gene. Primers were designed to screen for the H69‐V70 double deletion and N501Y mutations.

Samples were reverse‐transcribed, and PCR amplified from the same RNA samples used for the TaqPath assay using the One‐Step RT‐PCR kit (Qiagen). A nested PCR was performed with primers designed to identify the Δ69‐70 and N501Y mutations using the FastStart PCR Master (Sigma). Mutations were confirmed using agarose gel electrophoresis and successfully amplified samples were purified. The BigDye Xterminator kit v3.1 (Thermo‐Fisher) was used to purify the samples. A mix of 6.5 μl of highly deionized formamide with 3.5 μl of the purified product was used for capillary electrophoresis using a 3730xl DNA Analyzer. Electhropherogram data were aligned, and quality screened to their respective region using web RECall (beta v3.05). The two reference sequences used encompass codons 30–150 and codons 417–516 of the SARS‐CoV‐2 Spike gene. The consensus sequences were downloaded and aligned using MegaX software.

## 8 BIOSTATISTICS

Real‐time PCR data were analyzed, interpreted, and exported as.csv files using Applied Biosystems COVID‐19 Interpretive Software (version 1.3). *Ct* values from pooled samples were removed from the data set before the analysis. Scatter plots and boxplots were prepared to visualize *Ct* values data. Data were summarized and correlation analyses were performed. R (version 4.0.3) was used for biostatistics analyses and data visualization. Secondary data analysis was performed on data downloaded from GISAID.

## 9 RESULTS

**Figure 1:**
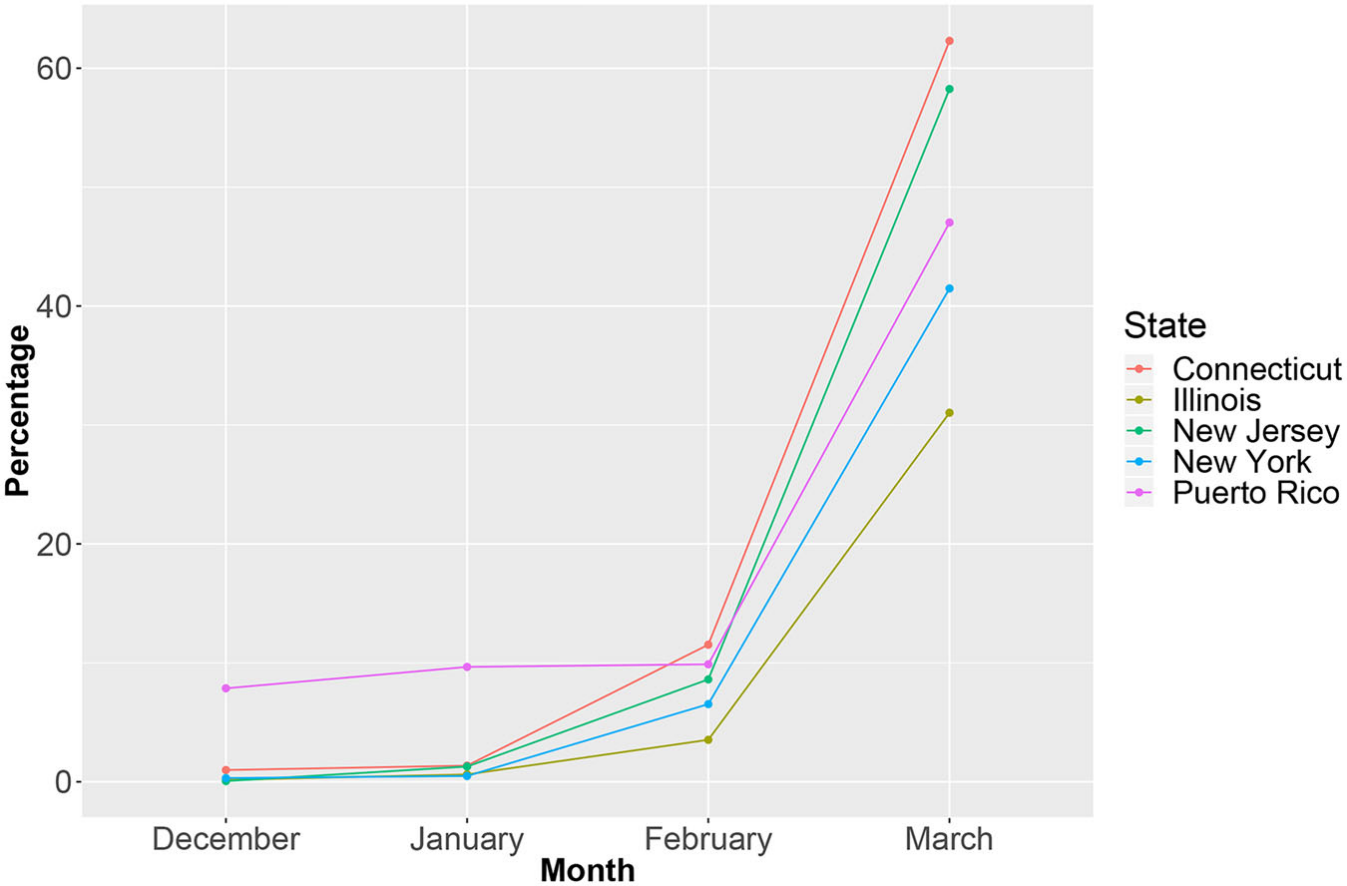
Frequency of S gene target failure (SGTF) in samples from Connecticut, Illinois, New Jersey, New York, and Puerto Rico from December 2020 through March 2021.

TaqPath data from close to 508,969 patients revealed a frequency of SGTF 47% for the last week of March 2021 in both Puerto Rico and US laboratories. SGTF steadily increased exponentially from 4% in November 2020 to 47% in March 2021 in Puerto Rico (Supporting Information: Figure 2A). The overall frequency of SGTL (15.1%), SGTF (9.2%), and SGTF with *N* and *ORF1ab* Cts <28 (2.5%) in Puerto Rico was high from March 2020 through March 2021. Ct values scatterplots reveal a complex relationship between *S, ORF1ab*, and *N*, for all values of *S* (Supporting Information: Figure 2A as well as between *S*and *ORF1ab, N*, and *MS2*, when *S* >= 33 (Supporting Information: Figure 2B). A bimodal distribution of *S Ct* values is clearly apparent when comparing box plots of *ORF1ab, N*, and *S*, for all values of *S*(Supporting Information: Figure 3A) with boxplots of *ORF1ab, N, MS2*, and *S*, when *S Ct* >=33 (Supporting Information: Figure 3B). The average weekly SGTF rate in US samples was high (>8%) from late December to early January, and then also increased exponentially through April (48%) (Figure 1). In January 2021, 47% of the SGTF samples were identified as Alpha when sequenced. In February, most (90%) of the sequenced samples were Alpha, including 100% (147/147) from February 15–23.^17^

A subset of samples (*n* = 100) with SGTF (74%) or high viral load in *S* (<28) were evaluated with Sanger sequencing (Supporting Information: Table 1). Most samples (97%) had a Spike mutation: ∆69/70 (91%), N501Y (91%), and 82% had both ∆69/70 and N501Y. A majority of the samples (58%) with *S* < 28 had the E484K mutation> E484K, a mutation located in the RBD region, is seen in SARS‐CoV‐2 B.1.351, P.1, P.2, and R.1 lineages.^32^

**Figure 2:**
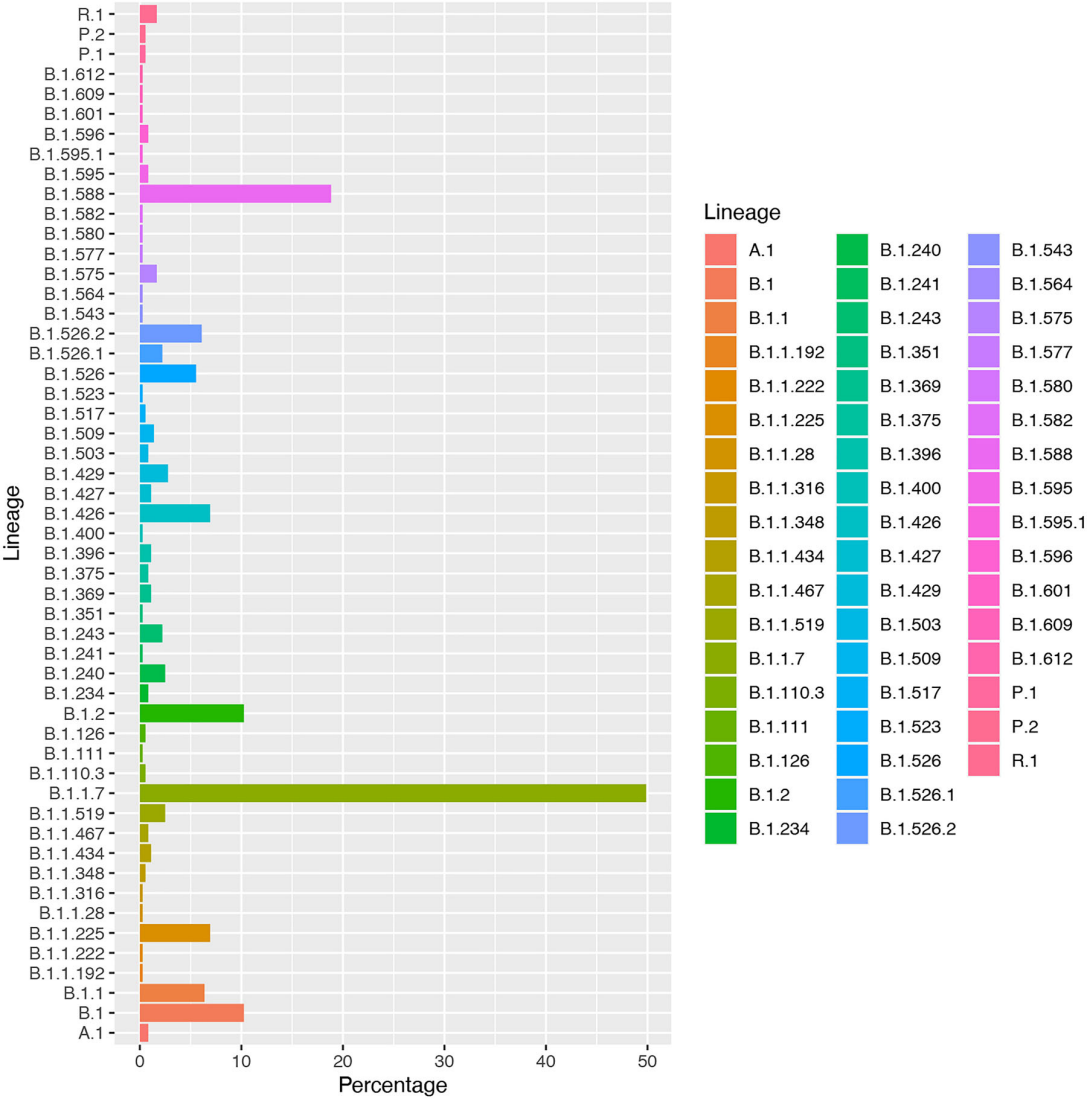
Frequency of SARS‐CoV‐2 variants in samples from Puerto Rico in GISAID, as of April 18, 2021 (*n* = 461), by lineage. GISAID, Global Influenza Surveillance and Response System; SARS‐CoV‐2, severe acute respiratory syndrome coronavirus 2.

The rise of SGFT in Puerto Rico is concurrent with a sharp increase in COVID‐19 variants identified among viral sequences from Puerto Rico uploaded to GISAID (Figure 2) as of April 18, 2021 (*n* = 461). Viral sequences from Puerto Rico are classified under 52 different lineages and 6 clades. Most samples were from nasopharyngeal specimens (73.9%) and are classified under seven distinct lineages: B.1.1.7 (27.7%%), B.1.588 (18.8%), B.1 (10%), B.1.2 (9.7%); B.1.1.225 (6.9%), B.1.426 (6.9%), and B.1.1 (6.4%) and four Clades: GH (49.7%), GR (21.5%), G (16.5%), and GRY (11.1%). Alpha frequency increased from <1% in the last week of January 2021 to 51.5% of viral sequences in the last week of March 2021, to become the dominant VOC in Puerto Rico.

**Figure 3:**
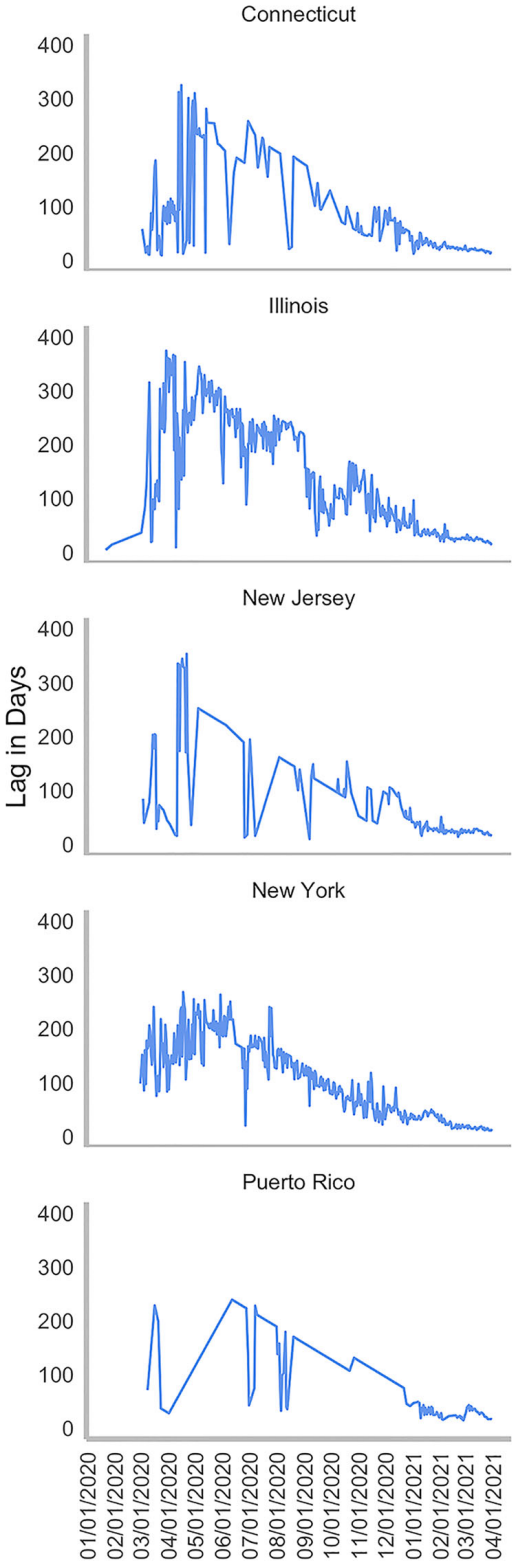
Mean daily lag time between sample collection and sequencing date from February 2020 through March 31, 2021, in Connecticut, Illinois, New Jersey, New York, and Puerto Rico.

There were 43,202 viral sequences from Connecticut, Illinois, New Jersey, New York, and Puerto Rico submitted to GISAID, as of March 31, 2021. The median lag‐time between sample collection and sample submission dates was 24 days. The lag‐time mean was 51 days. The minimum lag‐time was 3 days. The average daily lag‐time steadily declined from a mean of 17 days on March 1, 2021, to 14 days on March 31, 2021 (Figure 3).

**Figure 4:**
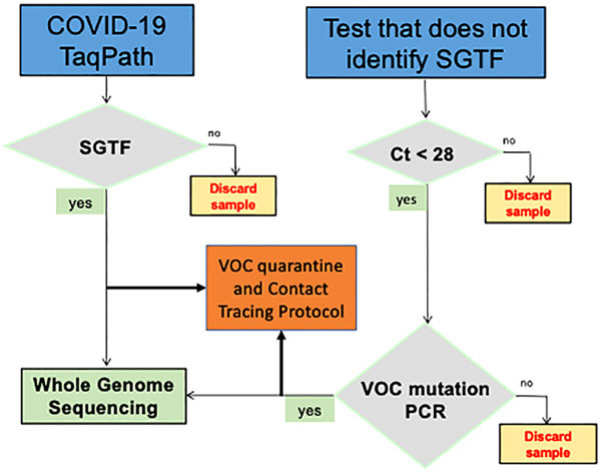
Variant of Concern Identification Algorithm diagram.

Summary statistics and Interquartile Range of *ORF1ab, N, S*, and *MS2* Cycle Threshold (*Ct*)^19^ values for samples from Puerto Ricolisted in Table 1, were used to develop a variant identificationalgorithm (Supporting Information: Materials). The commonly used formula to calculate outliers (Q1‐ (1.5 x IQR) was used to identify an evidence‐based *Ct* cutoff (*Ct* < 28) for a proposed VOC variant identification algorithm. A diagram of the proposed Variant of Concern Identification Algorithm summarizes the flow of samples from clinical laboratories where COVID‐19 PCR tests are performed, to laboratories where WGS is performed (Figure 4). Supporting Information: Figure 4 shows the parameters, decision‐making criteria, and timeline for each decision‐making node for clinical laboratories at community and regional levels, to sequencing laboratories at regional, state, and national levels.

**Table 1:** Summary statistics and interquartile range of cycle threshold (*Ct*) values for *ORF1ab, N*, and *S* genes

Gene	Min	Q1	Median	Mean	Q3	Max	NA	IQR	Outliers
Puerto Rico patients (*n* = 7510)									
*ORF1ab*	5.05	19.62	27.71	25.66	31.41	39.92	116	11.79	2
*N*	5.09	20.73	28.19	26.14	31.51	39.87	95	10.78	5
*S*	8.66	20.24	27.86	26.14	31.67	39.97	690	11.43	3
*MS2*	13.53	23.69	24.59	24.9	25.71	39.93	152	2.02	21
Puerto Rico patients with *Ct* values for *S* >= 33 and *S* gene target failure (*n* = 1824)									
*ORF1ab*	5.05	31.97	33.38	31.91	34.77	39.17	100	2.8	28
*N*	5.09	31.94	33.16	31.75	34.3	39.87	63	2.36	28
*S*	33	33.84	34.83	35.25	36.2	39.97	690	2.36	30
*MS2*	13.53	23.82	24.64	24.78	25.68	38.83	15	1.86	21

Samples with SGTF should be sequenced. Samples with *Ct* values < 28 for *N, ORF1ab or E* in SARS‐CoV‐2 clinical samples without *S* gene data should be evaluated for the presence of VOCs with Research Only Use (ROU) kits that identify specific VOC mutations, as triage before WGS. Patients infected with VOCs should be effectively triaged for isolation, contact tracing, and follow‐up treatment purposes. Patients with SGTF, or positive PCR results for N501Y or E484K, should be immediately placed into quarantine and a VOC contact tracing protocol needs to be urgently set in motion. According to the proposed Variant of Concern identification algorithm, at the time of this study, we found that 50% of currently positive samples in Puerto Rico and the United States should be managed as potential Alpha carriers with VOC self‐quarantine and contact tracing protocols, while WGS confirms their lineage in genomic surveillance laboratories.

## 10 DISCUSSION

Viral pandemics, including ones caused by coronaviruses such as SARS‐CoV‐2, have occurred repeatedly over the last century.^33^, ^34^ SARS‐CoV‐2 was more infectious than SARS‐CoV and quickly overwhelmed private and public health‐care systems worldwide. The lack of accurate real‐time SARS‐CoV‐2 data created massive challenges to local and national viral infection containment efforts. The need of a well‐coordinated genomic surveillance network to track viral spread and evolution, became apparent during the initial months of the pandemic. The United States did not have a national genomic surveillance program with WGS capability in place when the SARS‐COV‐2 pandemic began, therefore, only a small fraction of all new SARS‐CoV‐2 cases were initially sequenced ad‐hoc. The importance of having WGS capability to detect SARS‐CoV‐2 became apparent as soon as new highly infectious variants that can evade natural and vaccine‐acquired immunity began to spread.

The Precision Health Diagnostic and Surveillance Network (PHx) created one of the first evidenced‐based genomic surveillance algorithms in the United States, combining RT‐PCR and sequencing technologies to identify highly infectious SARS‐CoV‐2 VOCs. SGTF was used as an RT‐PCR proxy to monitor SARS‐CoV‐2 lineage prevalence and geo‐temporal distribution. PHx and similar algorithms have proven to be powerful tools to curb the spread of SARS‐CoV‐2 VOCs, enabling a reduction in illness, hospitalization, death, and post‐acute sequelae of COVID‐19 infection. Their use became critical, as highly infectious SGTF‐positive VOCs carrying mutations in the amino‐terminal domain of the Spike protein continue to emerge, each independent of previous variants.

The experience of Washington State Department of Health (WA DOH) is illustrative of the combined use of PCR and sequencing technologies to track the emergence and community spread of SARS‐CoV‐2 VOCs. Many laboratories share SGTF data with WA DOH, because WA DOH sequencing tracking process can take up to a few weeks. The coordination with private clinical laboratories has enabled WA DOH to rapidly track the spread of the highly infectious and vaccine‐resistant omicron VOC and inform public health action. Approximately 95% of the SGTF cases identified between December 20, 2021 and March 28, 2022 by clinical laboratories have been subsequently confirmed as omicron by WA DOH coordinated WGS sequencing. (https://doh.wa.gov/sites/default/files/2022-02/420-316-SequencingAndVariantsReport.pdf).

SARS‐CoV‐2 highly contagious VOCs have quickly spread around the world via multiple transmission modes, including, but not limited to, respiratory droplets, physical contact, and aerosols.^35^ The SARS‐CoV‐2 pandemic has also shown that household transmission, living density, and viral load, also are determinants of illness, hospitalization, death, and post‐acute sequelae of COVID‐19 infection.^36^, ^37^, ^38^, ^39^ Tracking SGTF cases can help scientists define community‐specific modes of SARS‐CoV‐2 transmission and rapidly implement effective surveillance and preventive measures designed to contain and quickly identify viral outbreaks in closed spaces, where large numbers of people congregate to travel, work, live, or play.^40^, ^41^

The SARS‐CoV‐2 pandemic, as previous pandemics, exhibits large clinical variability between individuals in the course of infection, ranging from asymptomatic infections to life‐threatening disease.^42^, ^43^ Inborn errors of, and autoantibodies directed against, type I interferons (IFNs) account for about 20% of critical COVID‐19 cases among SARS‐CoV‐2‐infected individuals.^44^, ^45^, ^46^ Individuals also show differences in response to SARS‐COV‐2 vaccination regimens in terms of vaccine efficiency, duration of immunity, and adverse effects.^47^, ^48^, ^49^, ^50^ Tracking SGTF cases can help health‐care administrators and public health policy makers plan for the care of vulnerable patients who can become critically ill. Adequate planning during the initial contagious phase of a new VOC can ensure that a sufficient number of hospital beds, treatments, and staff are in place before hospitalizations of critically ill patients begin to increase. Public health containment measures can also be quickly implemented in communities that really need them, because SGTF positive cases are on the rise.

Highly sensitive and specific molecular PCR tests, proven to be crucial to the COVID‐19 pandemic response, are recommended by WHO to confirm COVID‐19 diagnosis and activate public health measures.^51^, ^52^ The evidence we gathered from four different US States and Puerto Rico suggests that SGTF highly correlates with Alpha prevalence. The frequency of SGTF in Puerto Rico steadily increased from 4% in November 2020 to 47% in March 2021. Similarly, SGTF in the four US States, which was high (>8%) in early January, increased to 48% in March. Concurrently, Alpha became the dominant VOC in Puerto Rico and the United States in March 2021. Given the exponential rise in SGTF prevalence, a robust VOC genomic surveillance strategy must be quickly implemented. RT‐PCR can be used to identify potential VOC carriers. Patients with SGTF should be sequenced to identify viral lineage. Laboratories that do not amplify *S* can use ROU PCR or Sanger sequencing‐based alternatives to identify VOC's sentinel mutations in samples with *E, ORF1ab*, or *N Ct* values < 28, as a WGS triage strategy. Patients identified as presumptive carriers of a VOCs, by SGTF or *Ct* values < 28, should be placed in immediate preventive quarantine until WGS data confirms their variant status.

According to our evidence‐based algorithm, approximately 50% of all positive patients in the four States and Puerto Rico had SGTF in March 2021. At the time, the average lag time between clinical diagnosis and receipt of WGS results was approximately 15 days, SGTF patients were recommended to be placed immediately placed in self‐quarantine for a minimum of 14 days and required to have a negative PCR test result from a self‐collected sample at home before they could return to work or school. In parallel, VOC contact tracing efforts were recommended to be put in place, to prevent greater VOC community spread. Not all emerging SARS‐CoV‐2 variants demonstrate SGTF, and therefore this workflow would apply to SGTF‐correlated variants such as Alpha and Omicron.

Scientist in Portland Oregon used SGTF with *N* gene cycle threshold (*Ct*) < 30 as a proxy for lineage to monitor the early growth rate of Alpha and Omicron by calculating case doubling time. Lineage designation was later supported by viral genome sequencing via NextSeq (Illumina), also performed on samples with *N* gene *Ct* < 30. The first sequencing‐confirmed Alpha was collected on December 29, 2020, the first Delta on April 15, 2021, and the first Omicron on December 8, 2021, with SGTF appearing shortly after Alpha and Omicron. For comparison, Delta case doubling time was calculated using sequencing‐confirmed samples. Alpha had a case doubling time of 9.54 days based on 6,931 samples with SGTF. Delta's case doubling time was 19.30 days based on 447 sequenced samples. Omicron's case doubling time was 4.28 days, nearly half that of Alpha, based on 1,078 samples with SGTF.^53^

Genomic epidemiology tools that can quickly identify and track in real‐time COVID‐19 VOCs improve our understanding of the transmissibility, pathogenicity, morbidity, and mortality of each variant detected in geographically defined populations.^54^, ^55^ This approach will enable the deployment of targeted, evidence‐based strategies to quickly screen for COVID‐19 VOCs and identify clusters, leading to a decrease in the spread of community transmission. PHx, a critical consortium of researchers and scientists working in academia, industry, and clinical laboratories developed an evidence‐based method to screen SARS‐CoV‐2‐positive samples for COVID‐19 VOCs.

The PPM was a highly effective public health planning and evaluation framework to guide the conceptualization and implementation of PHx. Evaluation frameworks, such as PPM, can improve the understanding of the relationship between complex variables such as community attitudes, knowledge, and screening test utilization and implementation, determining the uptake of any screening intervention.^27^, ^56^ Given the complexity of behavioral change processes during a global pandemic such as COVID‐19, predisposing factors and barriers identified during the implementation of PHx can guide SARS‐CoV‐2 public policy and funding decision‐making. Lessons learned from PHx can inform the urgent deployment of precision health clinical and surveillance networks.

The information gathered by COVID‐19's precision health clinical and surveillance networks can be used to design community‐specific interventions to limit viral spread, and allocate community‐specific hospital beds and treatment doses required for the expected fraction of cases that will need hospitalization. SARS‐CoV‐2 variants have different clinical manifestations, with increased transmissibility, morbidity, and mortality of COVID‐19.^45^ VOC‐specific information should be considered in current practice and interventions to combat the pandemic and prevent related morbidity and mortality. VOC‐specific information gathered by precision health clinical and surveillance networks can also be used to monitor vaccine efficacy and adverse effects at the community level.^48^, ^49^

Using SGTF provides a real‐time estimate of viral epidemiology for Omicron but there are several limitations of this study. SGTF is not observed in SARS‐CoV‐2 sublineages of the Delta variant that are simultaneously in circulation.^57^ In addition, factors such as remote schooling, increased outdoor activities during summer, the protection from vaccination in the population, and the simultaneous presence of Alpha, Beta, and Gamma variants in circulation impacted the time it took for the Delta VOC to become a predominant variant. All of these may have contributed to Delta's prolonged case doubling time compared with that of Alpha and Omicron.^53^ However, the use of SGTF has been valuable in tracking highly infectious VOCs in which it is present. Other commercial PCR assays designed to track specific mutations associated with SGTF negative variants have been used by some clinical laboratories to identify potential VOCs. The lack of uniform workflows, reporting guidelines, and funding streams for the implementation and administration of a genomic surveillance network at national and state levels limited our ability to determine the impact of SGTF across the United States.

Some would argue that the proposed Variant of Concern Identification Algorithm usefulness is limited because it relies on the TaqPath COVID‐19 PCR test, which is prone to *S* gene failure. We argue that precisely because the TaqPath COVID‐19 PCR test is prone to SGTF, makes it a very useful tool to track highly contagious VOCs, such as Alpha and Omicron, which are prone to SGTF. Furthermore, the Variant of Concern Identification Algorithm includes the use of other commercially available PCR tests to identify VOCs in samples with high viral load (*Ct* < 28) originally tested with EUA‐approved PCR tests that are not prone to SGTF.

Finally, some may see as a limitation that the proposed Variant of Concern Identification Algorithm does not focus on the potential use of SGTF identification for therapeutic and vaccine development purposes. RNA viruses develop thousands of mutations as the number of replication cycles increase. RNA viruses with high mutation rates, such as SARS‐CoV‐2 may develop drug resistance and escape host natural, or vaccine‐acquired, immunity. SARS‐CoV‐2 has developed more than 4,000 mutations on the *S* gene alone.^58^ It is beyond the scope of this manuscript to examine the potential effect that SGTF variants and subvariants may have on host immunity or drug resistance. A genomic surveillance algorithm is a tool for rapid tracking highly infectious viral strains at local, regional, state, and national levels to provide evidence that supports public health and public policy measures to contain viral spread. It is a first line of defense to enable sound allocation of health‐care resources and staff to contain viral spread during pandemic and endemic stages of viral infections. However, WGS data produced by surveillance and clinical networks can eventually be used to develop COVID‐19 therapeutics and vaccines, to further contain the viral spread.

The convenience samples and data used for this report were gathered ad‐hoc by academic institutions, public and private organizations, and state and federal agencies. Data integrity, uniformity, and reliability are thus compromised, and should be treated as such. Uniform sample handling and management workflows, needed to assure data reproducibility, were not in place. For example, clinical laboratories discard their samples after diagnosis, which for COVID‐19 EUA‐approved tests, are qualitative decisions based on proprietary algorithms designed by test manufacturers. These closed PCR tests do not require *Ct* interpretation, nor molecular biology expertise either from the user. In addition, WGS is just entering the clinical and regulatory setting.

One of the most important lesson learned during the first year of the SARS‐CoV‐2 pandemic is that the business model of clinical laboratories, operating with small profit margins, does not have much leeway for collaborative clinical or research efforts. Therefore, clinical laboratory scientists and Department of Health staff do not have the resources and are not usually trained to sequence samples, analyze WGS data, or develop genomic surveillance programs based on RT‐PCR or WGS data. The combination of these complex factors, buttressed by sample and data management asymmetry between clinical and sequencing laboratories, as well as state and federal agencies, introduce barriers to standarization of sample and data workflows, eventually negatively impacting the  interpretation of SGTF and WGS resutls.

Our results suggest that a genomic surveillance network plays a critical role during the current stage of the COVID‐19 pandemic. Patients infected with VOCs should be secured into quarantine immediately, and VOC contact tracing efforts should be forcefully implemented to curtail community spread of VOCs. The evidence‐based Variant of Concern Identification algorithm developed by PHx can quickly detect emerging VOCs as a valuable tool for identifying individual carriers of highly infectious variants, who can then be effectively triaged for isolation, contact tracing, and treatment purposes. The reduced incidence of influenza and some other common respiratory infections during the SARS‐CoV‐2 pandemic suggests that sustainable and well‐operated precision health clinical and surveillance networks can be used to curtail highly contagious communicable diseases, today and in the future.^59^

## AUTHOR CONTRIBUTIONS

Rafael Guerrero‐Preston, Vanessa Rivera‐Amill, Karem Caraballo, Andrea Arias García, Raphael Sánchez Torres, Fernando Tadeu Zamuner, Matthew J. MacKay, Rachel Baits, Daisy Salgado, Gaurav Khullar, Jessica Metti, Timothy Baker, Joel Dudley, Jorge Acevedo Canabal, Josefina Romaguera, Ivonne Jiménez‐Velázquez, Adriana Baez, and Christopher E. Mason performed the research. Rafael Guerrero‐Preston designed the research study. Ana Purcell‐Wiltz, Keilyn Vale, Christopher E. Mason contributed essential reagents or tools. Rafael Guerrero‐Preston, Claudio Zanettini, and Luigi Marchionni analyzed the data.Rafael Guerrero‐Preston, Vanessa Rivera‐Amill, Sebastian Rodríguez‐Torres, Gabriela Pérez, José F. Rodríguez‐Orengo, and David Sidransky wrote and/or revised the paper. All authors have read and approved the final manuscript.

## CONFLICTS OF INTEREST

Matthew J. MacKay, Rachel Baits, Daisy Salgado, Gaurav Khullar, Jessica Metti, Timothy Baker, Joel Dudley, and Christopher E. Mason, work for Tempus Labs. Rafael Guerrero‐Preston works for LifeGene‐Biomarks. The remaining authors declare no conflict of interest.

## ETHICS STATEMENT

Approval from the University of Puerto Rico Medical School Institutional Review Board (IRB2770120) for a COVID‐19 biomarker development study was obtained to validate TaqPath COVID‐19 assay in paired, self‐collected nasal swab, saliva, and urine samples. Written informed consent was provided by the participants of the study.

## Supporting information

Supporting information.

Supporting information.

## Data Availability

Genomic data are available on GISAID repository: https://www.gisaid.org/ (see Supplemental S2 for accession numbers). RT‐PCR data that support the findings of this study are available from the corresponding author upon reasonable request.
