# Supplementary material for: Precision health diagnostic and surveillance network uses S gene target failure (SGTF) combined with sequencing technologies to track emerging SARS‐CoV‐2 variants
Source: Immun Inflamm Dis. 2022 May 11;10(6):e634. doi: 10.1002/iid3.634 (PMC9092005; doi:10.1002/iid3.634)
Supplement: Supplementary file 2 — Supporting information. [file IID3-10-0-s002.docx]

| **Supplemental_S2.** List of genomes from Puerto Rico used in the analysis. | |
| --- | --- |
| **GISAID ID** | **Originating lab** |
| EPI_ISL_447845 | PR - Biological and Chemical Emergencies Lab Office of Public Health Preparedness and Response |
| EPI_ISL_604719 | Quest Diagnostics |
| EPI_ISL_571899 | Quest Diagnostics |
| EPI_ISL_434541 | Puerto Rico Department of Health |
| EPI_ISL_434543 | Puerto Rico Department of Health |
| EPI_ISL_434542 | Puerto Rico Department of Health |
| EPI_ISL_434544 | Puerto Rico Department of Health |
| EPI_ISL_434545 | Puerto Rico Department of Health |
| EPI_ISL_434548 | Puerto Rico Department of Health |
| EPI_ISL_434549 | Puerto Rico Department of Health |
| EPI_ISL_434547 | Puerto Rico Department of Health |
| EPI_ISL_434550 | Puerto Rico Department of Health |
| EPI_ISL_434551 | Puerto Rico Department of Health |
| EPI_ISL_434546 | Puerto Rico Department of Health |
| EPI_ISL_434553 | Puerto Rico Department of Health |
| EPI_ISL_434552 | Puerto Rico Department of Health |
| EPI_ISL_940904 | Centers for Disease Control and Prevention, Dengue Branch |
| EPI_ISL_940907 | Centers for Disease Control and Prevention, Dengue Branch |
| EPI_ISL_940903 | Centers for Disease Control and Prevention, Dengue Branch |
| EPI_ISL_940912 | Centers for Disease Control and Prevention, Dengue Branch |
| EPI_ISL_940901 | Centers for Disease Control and Prevention, Dengue Branch |
| EPI_ISL_940900 | Centers for Disease Control and Prevention, Dengue Branch |
| EPI_ISL_513292 | University of Miami Immunology and Histocompatibility Laboratory |
| EPI_ISL_513293 | University of Miami Immunology and Histocompatibility Laboratory |
| EPI_ISL_539495 | Centers for Disease Control and Prevention, Dengue Branch |
| EPI_ISL_940905 | Centers for Disease Control and Prevention, Dengue Branch |
| EPI_ISL_1168694 | Puerto Rico Department of Health |
| EPI_ISL_940908 | Centers for Disease Control and Prevention, Dengue Branch |
| EPI_ISL_940909 | Centers for Disease Control and Prevention, Dengue Branch |
| EPI_ISL_940910 | Centers for Disease Control and Prevention, Dengue Branch |
| EPI_ISL_940911 | Centers for Disease Control and Prevention, Dengue Branch |
| EPI_ISL_940906 | Centers for Disease Control and Prevention, Dengue Branch |
| EPI_ISL_940902 | Centers for Disease Control and Prevention, Dengue Branch |
| EPI_ISL_940916 | Centers for Disease Control and Prevention, Dengue Branch |
| EPI_ISL_940919 | Centers for Disease Control and Prevention, Dengue Branch |
| EPI_ISL_940914 | Centers for Disease Control and Prevention, Dengue Branch |
| EPI_ISL_940913 | Centers for Disease Control and Prevention, Dengue Branch |
| EPI_ISL_940918 | Centers for Disease Control and Prevention, Dengue Branch |
| EPI_ISL_940917 | Centers for Disease Control and Prevention, Dengue Branch |
| EPI_ISL_940915 | Centers for Disease Control and Prevention, Dengue Branch |
| EPI_ISL_940928 | Centers for Disease Control and Prevention, Dengue Branch |
| EPI_ISL_940923 | Centers for Disease Control and Prevention, Dengue Branch |
| EPI_ISL_940922 | Centers for Disease Control and Prevention, Dengue Branch |
| EPI_ISL_940927 | Centers for Disease Control and Prevention, Dengue Branch |
| EPI_ISL_940920 | Centers for Disease Control and Prevention, Dengue Branch |
| EPI_ISL_940921 | Centers for Disease Control and Prevention, Dengue Branch |
| EPI_ISL_940925 | Centers for Disease Control and Prevention, Dengue Branch |
| EPI_ISL_940924 | Centers for Disease Control and Prevention, Dengue Branch |
| EPI_ISL_940933 | Centers for Disease Control and Prevention, Dengue Branch |
| EPI_ISL_940934 | Centers for Disease Control and Prevention, Dengue Branch |
| EPI_ISL_602328 | University of Miami Immunology and Histocompatibility Laboratory |
| EPI_ISL_940932 | Centers for Disease Control and Prevention, Dengue Branch |
| EPI_ISL_1168693 | Puerto Rico Department of Health |
| EPI_ISL_602326 | University of Miami Immunology and Histocompatibility Laboratory |
| EPI_ISL_605147 | University of Miami Immunology and Histocompatibility Laboratory |
| EPI_ISL_940929 | Centers for Disease Control and Prevention, Dengue Branch |
| EPI_ISL_940926 | Centers for Disease Control and Prevention, Dengue Branch |
| EPI_ISL_940930 | Centers for Disease Control and Prevention, Dengue Branch |
| EPI_ISL_940931 | Centers for Disease Control and Prevention, Dengue Branch |
| EPI_ISL_940935 | Centers for Disease Control and Prevention, Dengue Branch |
| EPI_ISL_602325 | University of Miami Immunology and Histocompatibility Laboratory |
| EPI_ISL_602327 | University of Miami Immunology and Histocompatibility Laboratory |
| EPI_ISL_1168695 | Puerto Rico Department of Health |
| EPI_ISL_527382 | University of Miami Immunology and Histocompatibility Laboratory |
| EPI_ISL_527384 | University of Miami Immunology and Histocompatibility Laboratory |
| EPI_ISL_527383 | University of Miami Immunology and Histocompatibility Laboratory |
| EPI_ISL_534693 | University of Miami Immunology and Histocompatibility Laboratory |
| EPI_ISL_527386 | University of Miami Immunology and Histocompatibility Laboratory |
| EPI_ISL_527385 | University of Miami Immunology and Histocompatibility Laboratory |
| EPI_ISL_940897 | Centers for Disease Control and Prevention, Dengue Branch |
| EPI_ISL_940756 | Centers for Disease Control and Prevention, Dengue Branch |
| EPI_ISL_940896 | Centers for Disease Control and Prevention, Dengue Branch |
| EPI_ISL_534694 | University of Miami Immunology and Histocompatibility Laboratory |
| EPI_ISL_534695 | University of Miami Immunology and Histocompatibility Laboratory |
| EPI_ISL_527388 | University of Miami Immunology and Histocompatibility Laboratory |
| EPI_ISL_527387 | University of Miami Immunology and Histocompatibility Laboratory |
| EPI_ISL_940895 | Centers for Disease Control and Prevention, Dengue Branch |
| EPI_ISL_527389 | University of Miami Immunology and Histocompatibility Laboratory |
| EPI_ISL_940899 | Centers for Disease Control and Prevention, Dengue Branch |
| EPI_ISL_940898 | Centers for Disease Control and Prevention, Dengue Branch |
| EPI_ISL_534696 | University of Miami Immunology and Histocompatibility Laboratory |
| EPI_ISL_534697 | University of Miami Immunology and Histocompatibility Laboratory |
| EPI_ISL_527396 | University of Miami Immunology and Histocompatibility Laboratory |
| EPI_ISL_534698 | University of Miami Immunology and Histocompatibility Laboratory |
| EPI_ISL_527390 | University of Miami Immunology and Histocompatibility Laboratory |
| EPI_ISL_527392 | University of Miami Immunology and Histocompatibility Laboratory |
| EPI_ISL_527393 | University of Miami Immunology and Histocompatibility Laboratory |
| EPI_ISL_527394 | University of Miami Immunology and Histocompatibility Laboratory |
| EPI_ISL_527395 | University of Miami Immunology and Histocompatibility Laboratory |
| EPI_ISL_527391 | University of Miami Immunology and Histocompatibility Laboratory |
| EPI_ISL_602324 | University of Miami Immunology and Histocompatibility Laboratory |
| EPI_ISL_527399 | University of Miami Immunology and Histocompatibility Laboratory |
| EPI_ISL_527397 | University of Miami Immunology and Histocompatibility Laboratory |
| EPI_ISL_527398 | University of Miami Immunology and Histocompatibility Laboratory |
| EPI_ISL_940951 | Centers for Disease Control and Prevention, Dengue Branch |
| EPI_ISL_940952 | Centers for Disease Control and Prevention, Dengue Branch |
| EPI_ISL_940953 | Centers for Disease Control and Prevention, Dengue Branch |
| EPI_ISL_940950 | Centers for Disease Control and Prevention, Dengue Branch |
| EPI_ISL_940959 | Centers for Disease Control and Prevention, Dengue Branch |
| EPI_ISL_940956 | Centers for Disease Control and Prevention, Dengue Branch |
| EPI_ISL_940955 | Centers for Disease Control and Prevention, Dengue Branch |
| EPI_ISL_940958 | Centers for Disease Control and Prevention, Dengue Branch |
| EPI_ISL_940957 | Centers for Disease Control and Prevention, Dengue Branch |
| EPI_ISL_940954 | Centers for Disease Control and Prevention, Dengue Branch |
| EPI_ISL_940968 | Centers for Disease Control and Prevention, Dengue Branch |
| EPI_ISL_940971 | Centers for Disease Control and Prevention, Dengue Branch |
| EPI_ISL_940972 | Centers for Disease Control and Prevention, Dengue Branch |
| EPI_ISL_942012 | Centers for Disease Control and Prevention, Dengue Branch |
| EPI_ISL_940969 | Centers for Disease Control and Prevention, Dengue Branch |
| EPI_ISL_940970 | Centers for Disease Control and Prevention, Dengue Branch |
| EPI_ISL_940963 | Centers for Disease Control and Prevention, Dengue Branch |
| EPI_ISL_940964 | Centers for Disease Control and Prevention, Dengue Branch |
| EPI_ISL_940965 | Centers for Disease Control and Prevention, Dengue Branch |
| EPI_ISL_940966 | Centers for Disease Control and Prevention, Dengue Branch |
| EPI_ISL_940967 | Centers for Disease Control and Prevention, Dengue Branch |
| EPI_ISL_942011 | Centers for Disease Control and Prevention, Dengue Branch |
| EPI_ISL_940973 | Centers for Disease Control and Prevention, Dengue Branch |
| EPI_ISL_940975 | Centers for Disease Control and Prevention, Dengue Branch |
| EPI_ISL_940974 | Centers for Disease Control and Prevention, Dengue Branch |
| EPI_ISL_942010 | Centers for Disease Control and Prevention, Dengue Branch |
| EPI_ISL_940962 | Centers for Disease Control and Prevention, Dengue Branch |
| EPI_ISL_942007 | Centers for Disease Control and Prevention, Dengue Branch |
| EPI_ISL_942009 | Centers for Disease Control and Prevention, Dengue Branch |
| EPI_ISL_940961 | Centers for Disease Control and Prevention, Dengue Branch |
| EPI_ISL_940987 | Centers for Disease Control and Prevention, Dengue Branch |
| EPI_ISL_940960 | Centers for Disease Control and Prevention, Dengue Branch |
| EPI_ISL_942008 | Centers for Disease Control and Prevention, Dengue Branch |
| EPI_ISL_940986 | Centers for Disease Control and Prevention, Dengue Branch |
| EPI_ISL_940983 | Centers for Disease Control and Prevention, Dengue Branch |
| EPI_ISL_940985 | Centers for Disease Control and Prevention, Dengue Branch |
| EPI_ISL_940984 | Centers for Disease Control and Prevention, Dengue Branch |
| EPI_ISL_940989 | Centers for Disease Control and Prevention, Dengue Branch |
| EPI_ISL_940990 | Centers for Disease Control and Prevention, Dengue Branch |
| EPI_ISL_940992 | Centers for Disease Control and Prevention, Dengue Branch |
| EPI_ISL_940991 | Centers for Disease Control and Prevention, Dengue Branch |
| EPI_ISL_940988 | Centers for Disease Control and Prevention, Dengue Branch |
| EPI_ISL_940993 | Centers for Disease Control and Prevention, Dengue Branch |
| EPI_ISL_940978 | Centers for Disease Control and Prevention, Dengue Branch |
| EPI_ISL_940976 | Centers for Disease Control and Prevention, Dengue Branch |
| EPI_ISL_940977 | Centers for Disease Control and Prevention, Dengue Branch |
| EPI_ISL_940981 | Centers for Disease Control and Prevention, Dengue Branch |
| EPI_ISL_940982 | Centers for Disease Control and Prevention, Dengue Branch |
| EPI_ISL_940979 | Centers for Disease Control and Prevention, Dengue Branch |
| EPI_ISL_940980 | Centers for Disease Control and Prevention, Dengue Branch |
| EPI_ISL_940937 | Centers for Disease Control and Prevention, Dengue Branch |
| EPI_ISL_940938 | Centers for Disease Control and Prevention, Dengue Branch |
| EPI_ISL_940936 | Centers for Disease Control and Prevention, Dengue Branch |
| EPI_ISL_940939 | Centers for Disease Control and Prevention, Dengue Branch |
| EPI_ISL_940940 | Centers for Disease Control and Prevention, Dengue Branch |
| EPI_ISL_940941 | Centers for Disease Control and Prevention, Dengue Branch |
| EPI_ISL_940949 | Centers for Disease Control and Prevention, Dengue Branch |
| EPI_ISL_940947 | Centers for Disease Control and Prevention, Dengue Branch |
| EPI_ISL_940943 | Centers for Disease Control and Prevention, Dengue Branch |
| EPI_ISL_940948 | Centers for Disease Control and Prevention, Dengue Branch |
| EPI_ISL_940944 | Centers for Disease Control and Prevention, Dengue Branch |
| EPI_ISL_940946 | Centers for Disease Control and Prevention, Dengue Branch |
| EPI_ISL_940942 | Centers for Disease Control and Prevention, Dengue Branch |
| EPI_ISL_940945 | Centers for Disease Control and Prevention, Dengue Branch |
| EPI_ISL_940994 | Centers for Disease Control and Prevention, Dengue Branch |
| EPI_ISL_1168671 | Centers for Disease Control and Prevention, Dengue Branch |
| EPI_ISL_1168660 | Centers for Disease Control and Prevention, Dengue Branch |
| EPI_ISL_1168677 | Centers for Disease Control and Prevention, Dengue Branch |
| EPI_ISL_1168661 | Centers for Disease Control and Prevention, Dengue Branch |
| EPI_ISL_1168678 | Centers for Disease Control and Prevention, Dengue Branch |
| EPI_ISL_1168668 | Centers for Disease Control and Prevention, Dengue Branch |
| EPI_ISL_1168679 | Centers for Disease Control and Prevention, Dengue Branch |
| EPI_ISL_1168669 | Centers for Disease Control and Prevention, Dengue Branch |
| EPI_ISL_1168670 | Centers for Disease Control and Prevention, Dengue Branch |
| EPI_ISL_1168663 | Centers for Disease Control and Prevention, Dengue Branch |
| EPI_ISL_1168653 | Centers for Disease Control and Prevention, Dengue Branch |
| EPI_ISL_1168682 | Inno Diagnostics/Center for Research Resources, Ponce Medical School Foundation, Inc. |
| EPI_ISL_1168662 | Centers for Disease Control and Prevention, Dengue Branch |
| EPI_ISL_1168664 | Centers for Disease Control and Prevention, Dengue Branch |
| EPI_ISL_1168696 | Puerto Rico Department of Health |
| EPI_ISL_1168675 | Centers for Disease Control and Prevention, Dengue Branch |
| EPI_ISL_1168673 | Centers for Disease Control and Prevention, Dengue Branch |
| EPI_ISL_1168654 | Centers for Disease Control and Prevention, Dengue Branch |
| EPI_ISL_1168680 | Centers for Disease Control and Prevention, Dengue Branch |
| EPI_ISL_1168689 | Puerto Rico Department of Health |
| EPI_ISL_1168665 | Centers for Disease Control and Prevention, Dengue Branch |
| EPI_ISL_1168681 | Centers for Disease Control and Prevention, Dengue Branch |
| EPI_ISL_1168672 | Centers for Disease Control and Prevention, Dengue Branch |
| EPI_ISL_1168666 | Centers for Disease Control and Prevention, Dengue Branch |
| EPI_ISL_1168658 | Centers for Disease Control and Prevention, Dengue Branch |
| EPI_ISL_1168659 | Centers for Disease Control and Prevention, Dengue Branch |
| EPI_ISL_1168656 | Centers for Disease Control and Prevention, Dengue Branch |
| EPI_ISL_888415 | Labcorp |
| EPI_ISL_887771 | Labcorp |
| EPI_ISL_888095 | Labcorp |
| EPI_ISL_1029254 | Laboratory Corporation of America |
| EPI_ISL_1027852 | Laboratory Corporation of America |
| EPI_ISL_1029255 | Laboratory Corporation of America |
| EPI_ISL_888069 | Labcorp |
| EPI_ISL_1029760 | Laboratory Corporation of America |
| EPI_ISL_887881 | Labcorp |
| EPI_ISL_1029051 | Laboratory Corporation of America |
| EPI_ISL_887783 | Labcorp |
| EPI_ISL_1027668 | Laboratory Corporation of America |
| EPI_ISL_1016506 | University of Miami Immunology and Histocompatibility Laboratory |
| EPI_ISL_1168674 | Centers for Disease Control and Prevention, Dengue Branch |
| EPI_ISL_1016505 | University of Miami Immunology and Histocompatibility Laboratory |
| EPI_ISL_886807 | Labcorp |
| EPI_ISL_1168687 | Puerto Rico Department of Health |
| EPI_ISL_1016484 | University of Miami Immunology and Histocompatibility Laboratory |
| EPI_ISL_1016479 | University of Miami Immunology and Histocompatibility Laboratory |
| EPI_ISL_1016502 | University of Miami Immunology and Histocompatibility Laboratory |
| EPI_ISL_1016504 | University of Miami Immunology and Histocompatibility Laboratory |
| EPI_ISL_1016503 | University of Miami Immunology and Histocompatibility Laboratory |
| EPI_ISL_886293 | Labcorp |
| EPI_ISL_887020 | Labcorp |
| EPI_ISL_1016482 | University of Miami Immunology and Histocompatibility Laboratory |
| EPI_ISL_1016501 | University of Miami Immunology and Histocompatibility Laboratory |
| EPI_ISL_967232 | Helix/Illumina |
| EPI_ISL_886294 | Labcorp |
| EPI_ISL_886677 | Labcorp |
| EPI_ISL_1026374 | Laboratory Corporation of America |
| EPI_ISL_1026536 | Laboratory Corporation of America |
| EPI_ISL_1026779 | Laboratory Corporation of America |
| EPI_ISL_1016485 | University of Miami Immunology and Histocompatibility Laboratory |
| EPI_ISL_1016499 | University of Miami Immunology and Histocompatibility Laboratory |
| EPI_ISL_1016500 | University of Miami Immunology and Histocompatibility Laboratory |
| EPI_ISL_1016481 | University of Miami Immunology and Histocompatibility Laboratory |
| EPI_ISL_1016496 | University of Miami Immunology and Histocompatibility Laboratory |
| EPI_ISL_1016498 | University of Miami Immunology and Histocompatibility Laboratory |
| EPI_ISL_1016497 | University of Miami Immunology and Histocompatibility Laboratory |
| EPI_ISL_886169 | Labcorp |
| EPI_ISL_1026191 | Laboratory Corporation of America |
| EPI_ISL_1168690 | Puerto Rico Department of Health |
| EPI_ISL_1168691 | Puerto Rico Department of Health |
| EPI_ISL_1168692 | Puerto Rico Department of Health |
| EPI_ISL_1168686 | Puerto Rico Department of Health |
| EPI_ISL_1016483 | University of Miami Immunology and Histocompatibility Laboratory |
| EPI_ISL_886997 | Labcorp |
| EPI_ISL_886248 | Labcorp |
| EPI_ISL_1016495 | University of Miami Immunology and Histocompatibility Laboratory |
| EPI_ISL_1016494 | University of Miami Immunology and Histocompatibility Laboratory |
| EPI_ISL_1168667 | Centers for Disease Control and Prevention, Dengue Branch |
| EPI_ISL_1168688 | Puerto Rico Department of Health |
| EPI_ISL_1016487 | University of Miami Immunology and Histocompatibility Laboratory |
| EPI_ISL_1016486 | University of Miami Immunology and Histocompatibility Laboratory |
| EPI_ISL_1016491 | University of Miami Immunology and Histocompatibility Laboratory |
| EPI_ISL_1016492 | University of Miami Immunology and Histocompatibility Laboratory |
| EPI_ISL_1016480 | University of Miami Immunology and Histocompatibility Laboratory |
| EPI_ISL_1016493 | University of Miami Immunology and Histocompatibility Laboratory |
| EPI_ISL_1016490 | University of Miami Immunology and Histocompatibility Laboratory |
| EPI_ISL_967815 | Helix/Illumina |
| EPI_ISL_1168684 | Inno Diagnostics/Center for Research Resources, Ponce Medical School Foundation, Inc. |
| EPI_ISL_1168683 | Inno Diagnostics/Center for Research Resources, Ponce Medical School Foundation, Inc. |
| EPI_ISL_1021729 | Laboratory Corporation of America |
| EPI_ISL_1021418 | Laboratory Corporation of America |
| EPI_ISL_1020799 | Laboratory Corporation of America |
| EPI_ISL_1021287 | Laboratory Corporation of America |
| EPI_ISL_1020830 | Laboratory Corporation of America |
| EPI_ISL_1021053 | Laboratory Corporation of America |
| EPI_ISL_966698 | Helix/Illumina |
| EPI_ISL_1020644 | Laboratory Corporation of America |
| EPI_ISL_1020671 | Laboratory Corporation of America |
| EPI_ISL_1168676 | Centers for Disease Control and Prevention, Dengue Branch |
| EPI_ISL_1168655 | Centers for Disease Control and Prevention, Dengue Branch |
| EPI_ISL_1168657 | Centers for Disease Control and Prevention, Dengue Branch |
| EPI_ISL_1016489 | University of Miami Immunology and Histocompatibility Laboratory |
| EPI_ISL_1201486 | INNO Diagnostics Reference Laboratory |
| EPI_ISL_1030754 | Laboratory Corporation of America |
| EPI_ISL_1016488 | University of Miami Immunology and Histocompatibility Laboratory |
| EPI_ISL_1031850 | Laboratory Corporation of America |
| EPI_ISL_1032151 | Laboratory Corporation of America |
| EPI_ISL_1031033 | Laboratory Corporation of America |
| EPI_ISL_967033 | Helix/Illumina |
| EPI_ISL_1168685 | Inno Diagnostics/Center for Research Resources, Ponce Medical School Foundation, Inc. |
| EPI_ISL_1031241 | Laboratory Corporation of America |
| EPI_ISL_1081280 | Laboratory Corporation of America |
| EPI_ISL_1038641 | Laboratory Corporation of America |
| EPI_ISL_1109633 | INNO Diagnostics Reference Laboratory |
| EPI_ISL_1037989 | Laboratory Corporation of America |
| EPI_ISL_1037788 | Laboratory Corporation of America |
| EPI_ISL_1037769 | Laboratory Corporation of America |
| EPI_ISL_1163003 | Laboratory Corporation of America |
| EPI_ISL_1235676 | INNO Diagnostics Reference Laboratory |
| EPI_ISL_1235674 | INNO Diagnostics Reference Laboratory |
| EPI_ISL_1235675 | INNO Diagnostics Reference Laboratory |
| EPI_ISL_1235670 | INNO Diagnostics Reference Laboratory |
| EPI_ISL_1222018 | Laboratory Corporation of America |
| EPI_ISL_1272918 | PR Public Health Lab |
| EPI_ISL_1252846 | INNO Diagnostics Reference Laboratory |
| EPI_ISL_1235677 | INNO Diagnostics Reference Laboratory |
| EPI_ISL_1272916 | PR Public Health Lab |
| EPI_ISL_1272917 | PR Public Health Lab |
| EPI_ISL_1252844 | INNO Diagnostics Reference Laboratory |
| EPI_ISL_1252845 | INNO Diagnostics Reference Laboratory |
| EPI_ISL_1235668 | INNO Diagnostics Reference Laboratory |
| EPI_ISL_1235669 | INNO Diagnostics Reference Laboratory |
| EPI_ISL_1222278 | Laboratory Corporation of America |
| EPI_ISL_1222358 | Laboratory Corporation of America |
| EPI_ISL_1222359 | Laboratory Corporation of America |
| EPI_ISL_1272919 | PR Public Health Lab |
| EPI_ISL_1235671 | INNO Diagnostics Reference Laboratory |
| EPI_ISL_1235672 | INNO Diagnostics Reference Laboratory |
| EPI_ISL_1235673 | INNO Diagnostics Reference Laboratory |
| EPI_ISL_1445362 | Aegis Sciences Corporation |
| EPI_ISL_1445298 | Aegis Sciences Corporation |
| EPI_ISL_1319187 | Laboratory Corporation of America |
| EPI_ISL_1620536 | Puerto Rico Department of Health |
| EPI_ISL_1620609 | Puerto Rico Department of Health |
| EPI_ISL_1620594 | Puerto Rico Department of Health |
| EPI_ISL_1445480 | Aegis Sciences Corporation |
| EPI_ISL_1620546 | Puerto Rico Department of Health |
| EPI_ISL_1620596 | Puerto Rico Department of Health |
| EPI_ISL_1620605 | Puerto Rico Department of Health |
| EPI_ISL_1620555 | Inno Diagnostics/Center for Research Resources, Ponce Medical School Foundation, Inc. |
| EPI_ISL_1620611 | Puerto Rico Department of Health |
| EPI_ISL_1620612 | Puerto Rico Department of Health |
| EPI_ISL_1620613 | Puerto Rico Department of Health |
| EPI_ISL_1620547 | Puerto Rico Department of Health |
| EPI_ISL_1620610 | Puerto Rico Department of Health |
| EPI_ISL_1422137 | Laboratory Corporation of America |
| EPI_ISL_1422138 | Laboratory Corporation of America |
| EPI_ISL_1338814 | Laboratory Corporation of America |
| EPI_ISL_1338815 | Laboratory Corporation of America |
| EPI_ISL_1338816 | Laboratory Corporation of America |
| EPI_ISL_1620615 | Puerto Rico Department of Health |
| EPI_ISL_1620616 | Puerto Rico Department of Health |
| EPI_ISL_1620548 | Puerto Rico Department of Health |
| EPI_ISL_1620614 | Puerto Rico Department of Health |
| EPI_ISL_1620598 | Puerto Rico Department of Health |
| EPI_ISL_1620606 | Puerto Rico Department of Health |
| EPI_ISL_1620580 | Puerto Rico Department of Health |
| EPI_ISL_1620599 | Puerto Rico Department of Health |
| EPI_ISL_1620600 | Puerto Rico Department of Health |
| EPI_ISL_1620601 | Puerto Rico Department of Health |
| EPI_ISL_1620617 | Puerto Rico Department of Health |
| EPI_ISL_1620618 | Puerto Rico Department of Health |
| EPI_ISL_1620602 | Puerto Rico Department of Health |
| EPI_ISL_1550559 | Aegis Sciences Corporation |
| EPI_ISL_1479555 | Aegis Sciences Corporation |
| EPI_ISL_1620581 | Inno Diagnostics/Center for Research Resources, Ponce Medical School Foundation, Inc. |
| EPI_ISL_1338813 | Laboratory Corporation of America |
| EPI_ISL_1338812 | Laboratory Corporation of America |
| EPI_ISL_1516263 | PR Public Health Lab |
| EPI_ISL_1620549 | Puerto Rico Department of Health |
| EPI_ISL_1620550 | Puerto Rico Department of Health |
| EPI_ISL_1620552 | Puerto Rico Department of Health |
| EPI_ISL_1620620 | Puerto Rico Department of Health |
| EPI_ISL_1620619 | Puerto Rico Department of Health |
| EPI_ISL_1620597 | Puerto Rico Department of Health |
| EPI_ISL_1620540 | Inno Diagnostics/Center for Research Resources, Ponce Medical School Foundation, Inc. |
| EPI_ISL_1620541 | Inno Diagnostics/Center for Research Resources, Ponce Medical School Foundation, Inc. |
| EPI_ISL_1620542 | Inno Diagnostics/Center for Research Resources, Ponce Medical School Foundation, Inc. |
| EPI_ISL_1338620 | Laboratory Corporation of America |
| EPI_ISL_1620622 | Puerto Rico Department of Health |
| EPI_ISL_1620627 | Puerto Rico Department of Health |
| EPI_ISL_1620603 | Puerto Rico Department of Health |
| EPI_ISL_1620621 | Puerto Rico Department of Health |
| EPI_ISL_1620534 | Inno Diagnostics/Center for Research Resources, Ponce Medical School Foundation, Inc. |
| EPI_ISL_1620538 | Inno Diagnostics/Center for Research Resources, Ponce Medical School Foundation, Inc. |
| EPI_ISL_1620539 | Inno Diagnostics/Center for Research Resources, Ponce Medical School Foundation, Inc. |
| EPI_ISL_1516262 | PR Public Health Lab |
| EPI_ISL_1620535 | Puerto Rico Department of Health |
| EPI_ISL_1620625 | Puerto Rico Department of Health |
| EPI_ISL_1620551 | Puerto Rico Department of Health |
| EPI_ISL_1620553 | Puerto Rico Department of Health |
| EPI_ISL_1620623 | Puerto Rico Department of Health |
| EPI_ISL_1620604 | Puerto Rico Department of Health |
| EPI_ISL_1620537 | Puerto Rico Department of Health |
| EPI_ISL_1620624 | Puerto Rico Department of Health |
| EPI_ISL_1620595 | Puerto Rico Department of Health |
| EPI_ISL_1560138 | Aegis Sciences Corporation |
| EPI_ISL_1560157 | Aegis Sciences Corporation |
| EPI_ISL_1560247 | Aegis Sciences Corporation |
| EPI_ISL_1562334 | Aegis Sciences Corporation |
| EPI_ISL_1620543 | Inno Diagnostics/Center for Research Resources, Ponce Medical School Foundation, Inc. |
| EPI_ISL_1620626 | Puerto Rico Department of Health |
| EPI_ISL_1562806 | Aegis Sciences Corporation |
| EPI_ISL_1562815 | Aegis Sciences Corporation |
| EPI_ISL_1562817 | Aegis Sciences Corporation |
| EPI_ISL_1562818 | Aegis Sciences Corporation |
| EPI_ISL_1562849 | Aegis Sciences Corporation |
| EPI_ISL_1563605 | Aegis Sciences Corporation |
| EPI_ISL_1513097 | Infinity Biologix |
| EPI_ISL_1620556 | Inno Diagnostics/Center for Research Resources, Ponce Medical School Foundation, Inc. |
| EPI_ISL_1620557 | Inno Diagnostics/Center for Research Resources, Ponce Medical School Foundation, Inc. |
| EPI_ISL_1620558 | Inno Diagnostics/Center for Research Resources, Ponce Medical School Foundation, Inc. |
| EPI_ISL_1550781 | Aegis Sciences Corporation |
| EPI_ISL_1561459 | Aegis Sciences Corporation |
| EPI_ISL_1561468 | Aegis Sciences Corporation |
| EPI_ISL_1561651 | Aegis Sciences Corporation |
| EPI_ISL_1561650 | Aegis Sciences Corporation |
| EPI_ISL_1561509 | Aegis Sciences Corporation |
| EPI_ISL_1620566 | Inno Diagnostics/Center for Research Resources, Ponce Medical School Foundation, Inc. |
| EPI_ISL_1444416 | Quest Diagnostics Incorporated |
| EPI_ISL_1563334 | Aegis Sciences Corporation |
| EPI_ISL_1562888 | Aegis Sciences Corporation |
| EPI_ISL_1620559 | Inno Diagnostics/Center for Research Resources, Ponce Medical School Foundation, Inc. |
| EPI_ISL_1620582 | Inno Diagnostics/Center for Research Resources, Ponce Medical School Foundation, Inc. |
| EPI_ISL_1548622 | Laboratory Corporation of America |
| EPI_ISL_1563112 | Aegis Sciences Corporation |
| EPI_ISL_1620544 | Inno Diagnostics/Center for Research Resources, Ponce Medical School Foundation, Inc. |
| EPI_ISL_1620545 | Inno Diagnostics/Center for Research Resources, Ponce Medical School Foundation, Inc. |
| EPI_ISL_1620560 | Inno Diagnostics/Center for Research Resources, Ponce Medical School Foundation, Inc. |
| EPI_ISL_1620561 | Inno Diagnostics/Center for Research Resources, Ponce Medical School Foundation, Inc. |
| EPI_ISL_1620565 | Inno Diagnostics/Center for Research Resources, Ponce Medical School Foundation, Inc. |
| EPI_ISL_1620554 | Puerto Rico Department of Health |
| EPI_ISL_1563269 | Aegis Sciences Corporation |
| EPI_ISL_1620562 | Inno Diagnostics/Center for Research Resources, Ponce Medical School Foundation, Inc. |
| EPI_ISL_1620563 | Inno Diagnostics/Center for Research Resources, Ponce Medical School Foundation, Inc. |
| EPI_ISL_1620564 | Inno Diagnostics/Center for Research Resources, Ponce Medical School Foundation, Inc. |
| EPI_ISL_1620583 | Inno Diagnostics/Center for Research Resources, Ponce Medical School Foundation, Inc. |
| EPI_ISL_1620533 | Inno Diagnostics/Center for Research Resources, Ponce Medical School Foundation, Inc. |
| EPI_ISL_1551160 | Aegis Sciences Corporation |
| EPI_ISL_1551162 | Aegis Sciences Corporation |
| EPI_ISL_1551171 | Aegis Sciences Corporation |
| EPI_ISL_1551282 | Aegis Sciences Corporation |
| EPI_ISL_1491923 | Aegis Sciences Corporation |
| EPI_ISL_1513442 | Aegis Sciences Corporation |
| EPI_ISL_1620567 | Inno Diagnostics/Center for Research Resources, Ponce Medical School Foundation, Inc. |
| EPI_ISL_1620568 | Inno Diagnostics/Center for Research Resources, Ponce Medical School Foundation, Inc. |
| EPI_ISL_1620584 | Inno Diagnostics/Center for Research Resources, Ponce Medical School Foundation, Inc. |
| EPI_ISL_1620608 | Inno Diagnostics/Center for Research Resources, Ponce Medical School Foundation, Inc. |
| EPI_ISL_1491900 | Aegis Sciences Corporation |
| EPI_ISL_1557104 | Fulgent Genetics |
| EPI_ISL_1557105 | Fulgent Genetics |
| EPI_ISL_1620570 | Inno Diagnostics/Center for Research Resources, Ponce Medical School Foundation, Inc. |
| EPI_ISL_1620572 | Inno Diagnostics/Center for Research Resources, Ponce Medical School Foundation, Inc. |
| EPI_ISL_1620585 | Inno Diagnostics/Center for Research Resources, Ponce Medical School Foundation, Inc. |
| EPI_ISL_1620586 | Inno Diagnostics/Center for Research Resources, Ponce Medical School Foundation, Inc. |
| EPI_ISL_1620587 | Inno Diagnostics/Center for Research Resources, Ponce Medical School Foundation, Inc. |
| EPI_ISL_1620588 | Inno Diagnostics/Center for Research Resources, Ponce Medical School Foundation, Inc. |
| EPI_ISL_1620589 | Inno Diagnostics/Center for Research Resources, Ponce Medical School Foundation, Inc. |
| EPI_ISL_1528460 | PR Public Health Lab |
| EPI_ISL_1528461 | PR Public Health Lab |
| EPI_ISL_1551611 | Aegis Sciences Corporation |
| EPI_ISL_1560956 | Aegis Sciences Corporation |
| EPI_ISL_1513609 | Aegis Sciences Corporation |
| EPI_ISL_1620569 | Inno Diagnostics/Center for Research Resources, Ponce Medical School Foundation, Inc. |
| EPI_ISL_1620571 | Inno Diagnostics/Center for Research Resources, Ponce Medical School Foundation, Inc. |
| EPI_ISL_1620592 | Inno Diagnostics/Center for Research Resources, Ponce Medical School Foundation, Inc. |
| EPI_ISL_1552938 | Quest Diagnostics Incorporated |
| EPI_ISL_1552969 | Quest Diagnostics Incorporated |
| EPI_ISL_1552755 | Quest Diagnostics Incorporated |
| EPI_ISL_1556786 | Fulgent Genetics |
| EPI_ISL_1614440 | Fulgent Genetics |
| EPI_ISL_1620591 | Inno Diagnostics/Center for Research Resources, Ponce Medical School Foundation, Inc. |
| EPI_ISL_1610733 | Laboratory Corporation of America |
| EPI_ISL_1614204 | Fulgent Genetics |
| EPI_ISL_1614441 | Fulgent Genetics |
| EPI_ISL_1610995 | Laboratory Corporation of America |
| EPI_ISL_1610732 | Laboratory Corporation of America |
| EPI_ISL_1610994 | Laboratory Corporation of America |
| EPI_ISL_1620574 | Puerto Rico Department of Health |
| EPI_ISL_1620575 | Puerto Rico Department of Health |
| EPI_ISL_1620576 | Puerto Rico Department of Health |
| EPI_ISL_1620577 | Puerto Rico Department of Health |
| EPI_ISL_1620578 | Puerto Rico Department of Health |
| EPI_ISL_1620579 | Puerto Rico Department of Health |
| EPI_ISL_1620593 | Puerto Rico Department of Health |
| EPI_ISL_1620607 | Puerto Rico Department of Health |
| EPI_ISL_1620573 | Inno Diagnostics/Center for Research Resources, Ponce Medical School Foundation, Inc. |
| EPI_ISL_1620590 | Inno Diagnostics/Center for Research Resources, Ponce Medical School Foundation, Inc. |
| EPI_ISL_1610993 | Laboratory Corporation of America |
| EPI_ISL_1621987 | Quest Diagnostics Incorporated |
| EPI_ISL_1622023 | Quest Diagnostics Incorporated |
| EPI_ISL_1622082 | Quest Diagnostics Incorporated |
